# HOXC4 promotes proliferation of pancreatic cancer cells by increasing LDHA-mediated glycolysis

**DOI:** 10.18632/aging.206008

**Published:** 2024-07-09

**Authors:** Hao Zhang, Bing Han, She Tian, Yongjun Gong, Liwen Chen, Li Liu

**Affiliations:** 1 College of Clinical Medicine, Guizhou Medical University, Guiyang, China; 2 Department of Hepatic-Biliary-Pancreatic Surgery, The Affiliated Hospital of Guizhou Medical University, Guiyang, China; 3 Guizhou Provincial Key Laboratory of Pathogenesis and Drug Research on Common Chronic Diseases, Guizhou Medical University, Guiyang, China; 4 School of Public Health, Guizhou Medical University, Guiyang, China

**Keywords:** pancreatic cancer, HOXC4, lactate dehydrogenase A, cell proliferation, glycolysis

## Abstract

Homeobox C4 (HOXC4) is a member of homeobox family and acts as a transcription factor in regulating morphological development. The current study aimed to determine its role in pancreatic cancer (PC). Bioinformatics analysis was employed to assess the expression and clinical significance of HOXC4 in PC, while the expression of HOXC4 was further confirmed in PC tissues through quantitative real-time polymerase chain reaction (qRT-PCR) and immunohistochemistry (IHC). The impact of HOXC4 on PC cell proliferation was evaluated using various assays including Cell Counting Kit-8, colony formation, apoptosis detection, cell cycle analysis, and subcutaneous tumorigenesis. Extracellular acidification rate, glucose uptake, and lactate production measurements were detected to examine the impact of HOXC4 on glycolysis. The relationship between HOXC4 and lactate dehydrogenase A (LDHA) was investigated using CHIP assay, luciferase reporter assay, and western blot. Notably, there was a substantial increase in HOXC4 expression in PC, and patients with elevated HOXC4 levels exhibited shorter survival durations. HOXC4 knockdown resulted in significantly reduced proliferation and colony formation in PC cells, accompanied by increased apoptosis and G1 phase arrest. The overexpression of HOXC4 resulted in contrasting effects. *In vivo*, the proliferation of PC cells was diminished upon the knockdown of HOXC4. HOXC4 exhibited an increase in LDHA expression by binding to its promoter. The suppressive effects of HOXC4 knockdown on PC cells were counteracted upon the restoration of LDHA. In conclusion, HOXC4 promoted the proliferation of PC cells by increasing LDHA-mediated glycolysis. HOXC4 can act as a target for PC therapy.

## INTRODUCTION

Less than 5% of individuals achieve a five-year survival rate following the diagnosis of pancreatic cancer (PC) [1]. Patients with PC frequently experience early metastasis and rapid invasion. While curative resection serves as the primary therapeutic approach for PC, it is only feasible for a mere 15–20% of patients diagnosed at an early stage. Additionally, recurrences are most likely to transpire within a two-year period following resection [2, 3]. Enhanced comprehension of the molecular mechanisms implicated in the progression of PC may facilitate the diagnosis and treatment of this condition.

Aerobic glycolysis, known as the Warburg effect, is a prominent characteristic observed in various types of cancer, including PC [4, 5]. Cancer cells exhibit a preference for metabolizing glucose through glycolysis, even in oxygen-rich environments, rather than utilizing the tricarboxylic acid cycle. Previous studies have identified several key enzymes involved in regulating glycolysis, such as phosphofructokinase (PFKP), glucose transporter 1, and lactate dehydrogenase A (LDHA) [6, 7]. Numerous investigations have elucidated the impact of glycolysis on the development of PC [8]. Similarly, a previous study showed that increased glycolysis in PC cells promoted the drug-resistance of PC to gemcitabine [9]. As a key enzyme involved in glycolysis, LDHA was found to increase in PC tissues and associated with poor prognosis [10]. LDHA-induced glycolysis promoted PC cell proliferation and metastasis [11]. Moreover, LDHA also affected the microenvironment via inhibiting the function of NK cells mediated by lactate [12, 13]. Therefore, identification of the regulation mechanism of LDHA-induced glycolysis may help the therapy of PC.

Homeobox (HOX) genes, ubiquitous in nearly all eukaryotes, exert their regulatory influence by encoding transcription factors [14]. HOXC4 has been identified as a member of the HOX family and has been found to be upregulated in various cancer types, including PC [15] and uveal melanoma [16]. Additionally, elevated levels of HOXC4 have been observed in gastric cancer, where they have been associated with a poor prognosis [17]. Moreover, HOXC4 has been shown to enhance the mobility of hepatocellular carcinoma cells through the transactivation of Snail [18]. However, the specific effects of HOXC4 in PC remain poorly understood.

This study elucidated the role and mechanisms of HOXC4 in PC by conducting a series of biological function and molecular experiments. The findings suggest that HOXC4 may serve as a significant biomarker for the diagnosis and treatment of PC, as it was found to enhance the proliferation of PC cells through the upregulation of LDHA-mediated glycolysis.

## RESULTS

### High expression of HOXC4 was observed in PC tissues, and predicted poor outcome

**Figure 1:**
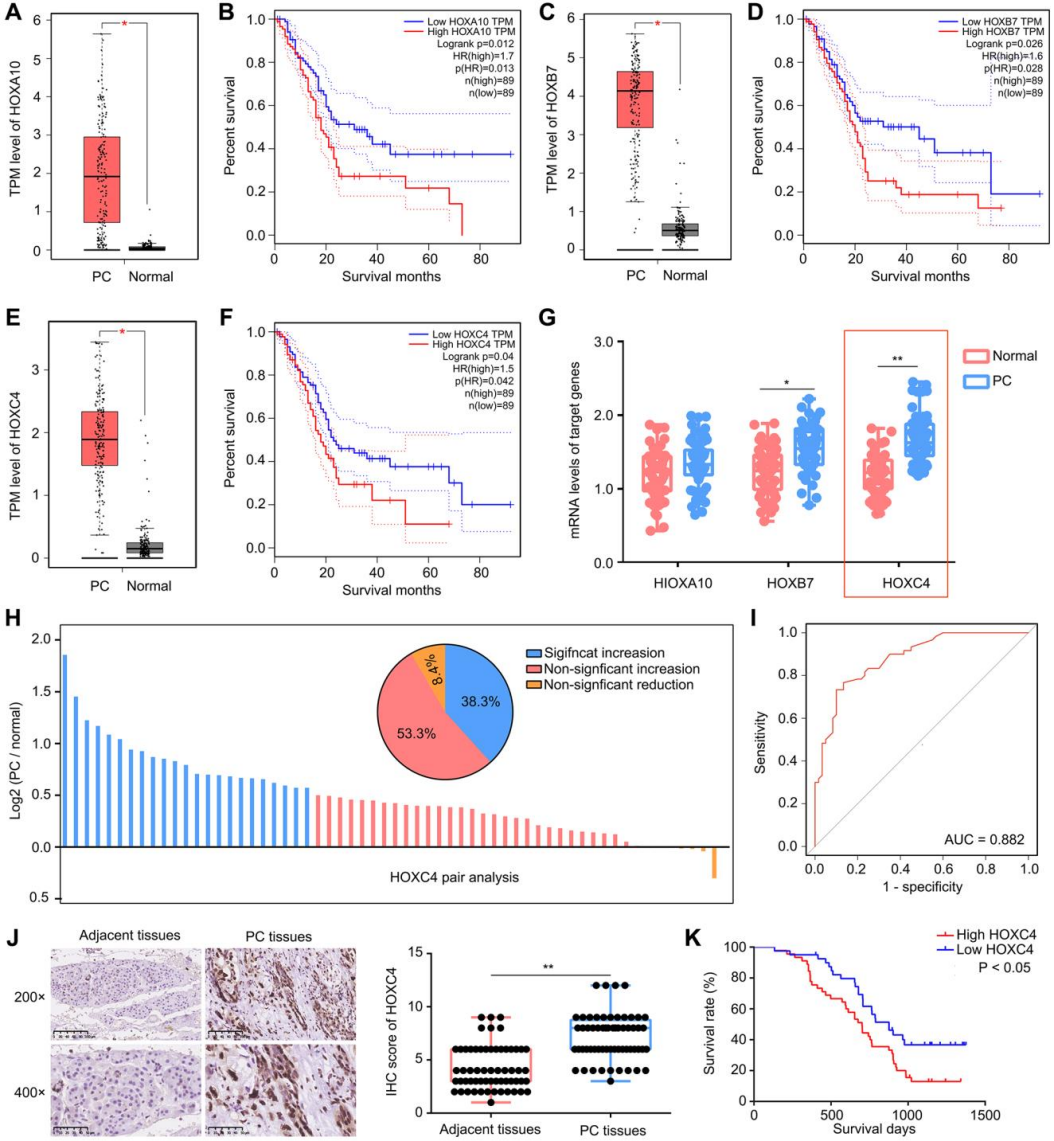
**High expression of HOXC4 was observed in PC tissues and predicted poor outcome.** (**A**, **B**) HOXA10 was highly expressed in PC tissues and associated with lower overall survival days. (**C**, **D**) HOXB7 was highly expressed in PC tissues and associated with lower overall survival days. (**E**, **F**) HOXC4 was highly expressed in PC tissues and associated with lower overall survival days. (**G**) qRT-PCR was used to detect the expression of HOXA10, HOXB7 and HOXC4 in PC tissues and adjacent tissues. (**H**) Pair analysis indicated that 53.5% PC patients had increased expression of HOXC4. (**I**) Diagnostic value of HOXC4 in PC. (**J**) IHC was used to detect the expression of HOXC4 in PC tissues and adjacent tissues. (**K**) KM plot indicated the survival days of PC patients with high and low HOXC4 expression. ^*^P < 0.05; ^**^P < 0.01.

In previous researches, it has been established that HOX family genes are dysregulated in different types of cancers. In this study, we first conducted an analysis of the expression patterns of HOX genes (Supplementary Table 1) in PC tissues using data from the TCGA database. Our analysis revealed that three members of the HOX family, namely HOXA10 (Figure 1A, 1B), HOXB7 (Figure 1C, 1D), and HOXC4 (Figure 1E, 1F), exhibited elevated expression levels in PC tissues compared to adjacent tissues and positively correlated with a lower overall survival rate. Based on these findings, we decided to focus our investigation on these three HOX family members and further validated their expression in PC tissues from our research cohort. Based on the qRT-PCR results, it was observed that HOXC4 demonstrated the most substantial increase in PC tissues within the research cohort (Figure 1G). Furthermore, upon conducting pair analysis, it was determined that 53.3% of PC patients exhibited a significant elevation (expression in PC/ normal ≥1.5) in HOXC4 expression (Figure 1H). The diagnostic efficacy of HOXC4 mRNA levels in distinguishing PC and adjacent tissues was calculated to be 0.882 (Figure 1I). Similarly, a higher protein level of HOXC4 was detected in PC tissues compared to adjacent tissues (Figure 1J). Moreover, our investigation revealed a significant correlation between elevated levels of HOXC4 mRNA expression in patients with PC and a decreased overall survival rate (Figure 1K). In light of these findings, it can be inferred that HOXC4 potentially functions as an oncogene in PC and is linked to an unfavorable prognosis.

### HOXC4 regulates PC cell proliferation, cell apoptosis and G1 phase arrest *in vitro*

**Figure 2:**
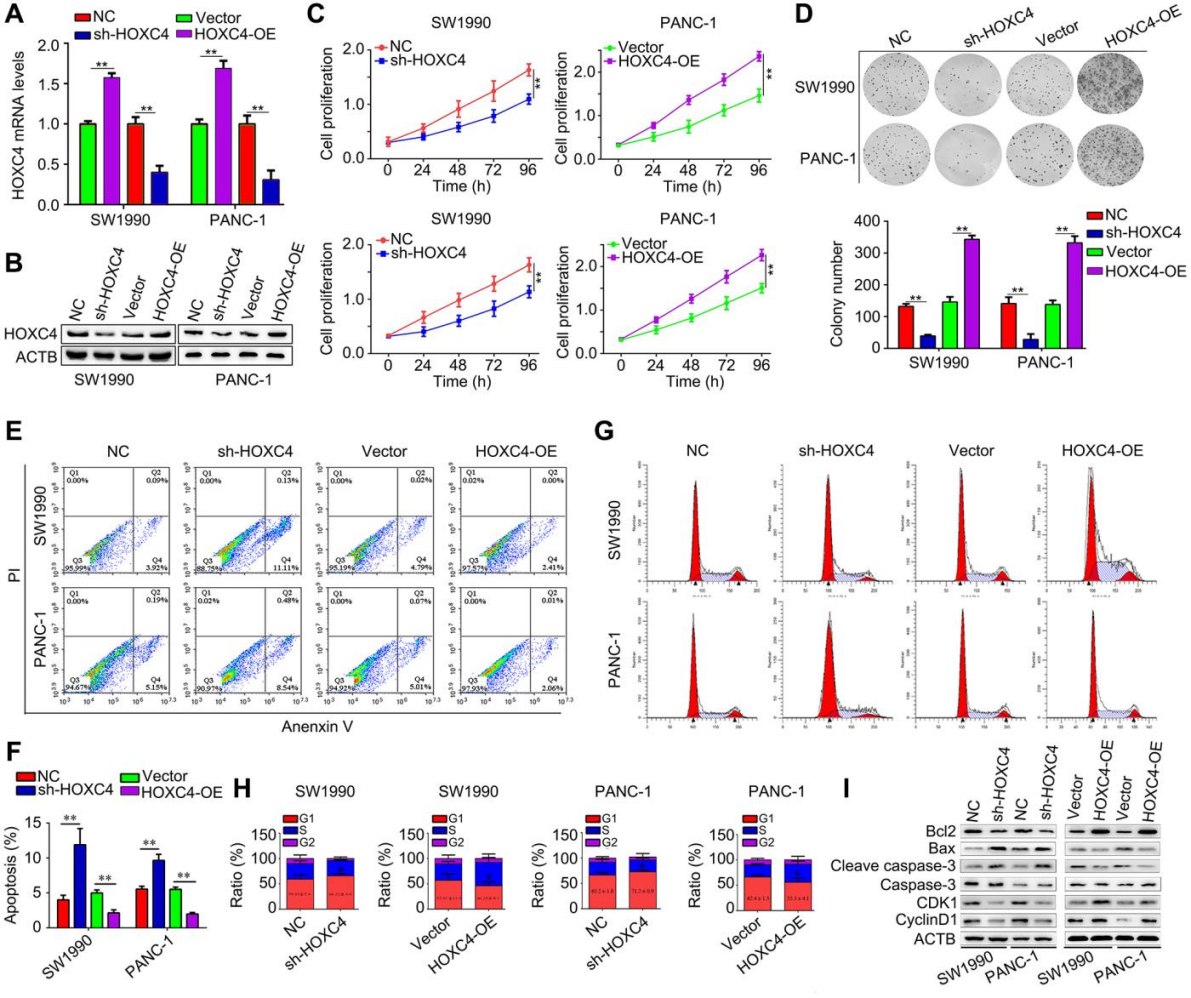
**HOXC4 regulates PC cell proliferation, apoptosis and cell cycle distribution *in vitro*.** (**A**, **B**) The transfection efficiency of HOXC4 overexpression lentivirus (HOXC4-OE) and HOXC4 shRNAs (sh-HOXC4). (**C**) Cell counting kit-8 assay was used to detect the effect of HOXC4 on PC cell proliferation. (**D**) Colony formation assay was used to detect the effect of HOXC4 on PC cell colony formation. (**E**, **F**) Flow cytometry was used to detect the effect of HOXC4 on PC cell apoptosis. (**G**, **H**) Flow cytometry was used to detect the effect of HOXC4 on the cell cycle distribution of PC cells. (**I**) Western blotting was used to detect the expression of Bcl-2, Bax, caspase 3, cleaved caspases 3, cyclin-dependent kinase 1 and cyclin D1 while HOXC4 was overexpressed and inhibited. ^**^P < 0.01.

Lentivirus for overing HOXC4, shRNAs targeting HOXC4 and their control were transfected to affect the expression level of HOXC4 and determine its biological function (Figure 2A, 2B). HOXC4 knockdown significantly decreased PC proliferation, while HOXC4 overexpression increased it (Figure 2C). In addition, HOXC4 knockdown decreased colony formation, while overexpression of HOXC4 increased the number of colonies (Figure 2D). Knockdown of HOXC4 significantly increased apoptosis in PC cells, while increasing the expression of HOXC4 decreased cell apoptosis (Figure 2E, 2F). Similarly, as shown by cell cycle distribution analysis, HOXC4 knockdown significantly increased G1-phase PC cells, while number of G1-phase cells was decreased when HOXC4 was overexpressed (Figure 2G, 2H). Furthermore, several biomarkers of apoptosis and cell cycle, including Bcl-2, Bax, caspase 3, cleaved caspase-3, CDK1 and cyclin D1, were detected. According to western blotting, the HOXC4 knockdown group cells showed significantly reduced levels of Bcl-2, CDK1 and cyclin D1, while cleaved caspase 3 and Bax expression were significantly elevated. HOXC4 overexpression increased the expression of Bcl-2, CDK1 and cyclin D1, while decreasing Bax and caspase-3 expression (Figure 2I).

### Knockdown of HOXC4 suppresses *in vivo* proliferation of PC cells

**Figure 3:**
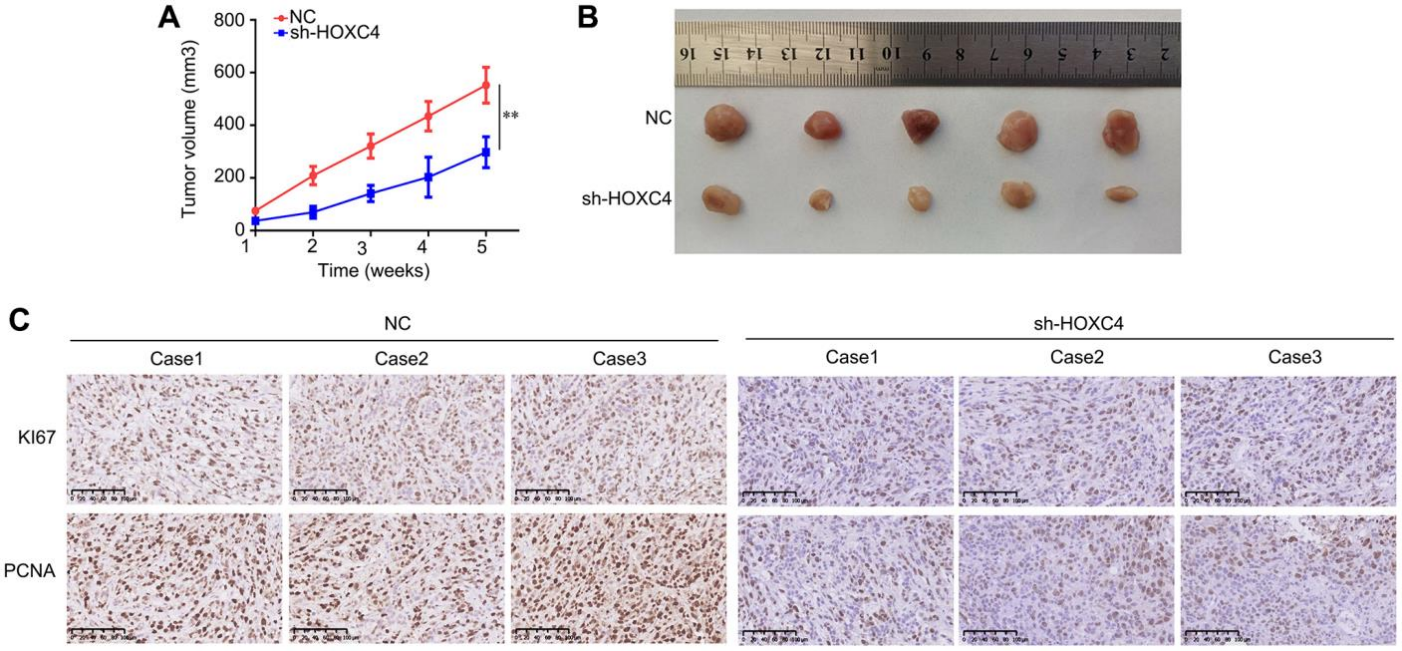
**HOXC4 knockdown inhibits the proliferation of PANC-1 cells *in vivo*.** (**A**) Growth curve of the tumors in the HOXC4 knockdown and control groups. (**B**) Images of the tumors. (**C**) Expression of Ki67 and proliferating cell nuclear antigen in HOXC4 knockdown and normal control tissues. ^**^P < 0.01.

We conducted an assessment of the impact of HOXC4 on the proliferation of PC cells *in vivo* by employing a subcutaneous tumorigenesis model. Specifically, we administered subcutaneous injections of HOXC4 knockdown and normal PANC-1 cells into the right upper flank of mice. The findings demonstrated that the growth rate of PC tissues with HOXC4 knockdown was significantly slower compared to that of normal cells (Figure 3A, 3B). Immunohistochemical analysis further revealed that PC tissues with HOXC4 knockdown exhibited notably reduced expression levels of PCNA and Ki67 in comparison to the NC group (Figure 3C).

### HOXC4 regulates the glycolysis of PC cells

**Figure 4:**
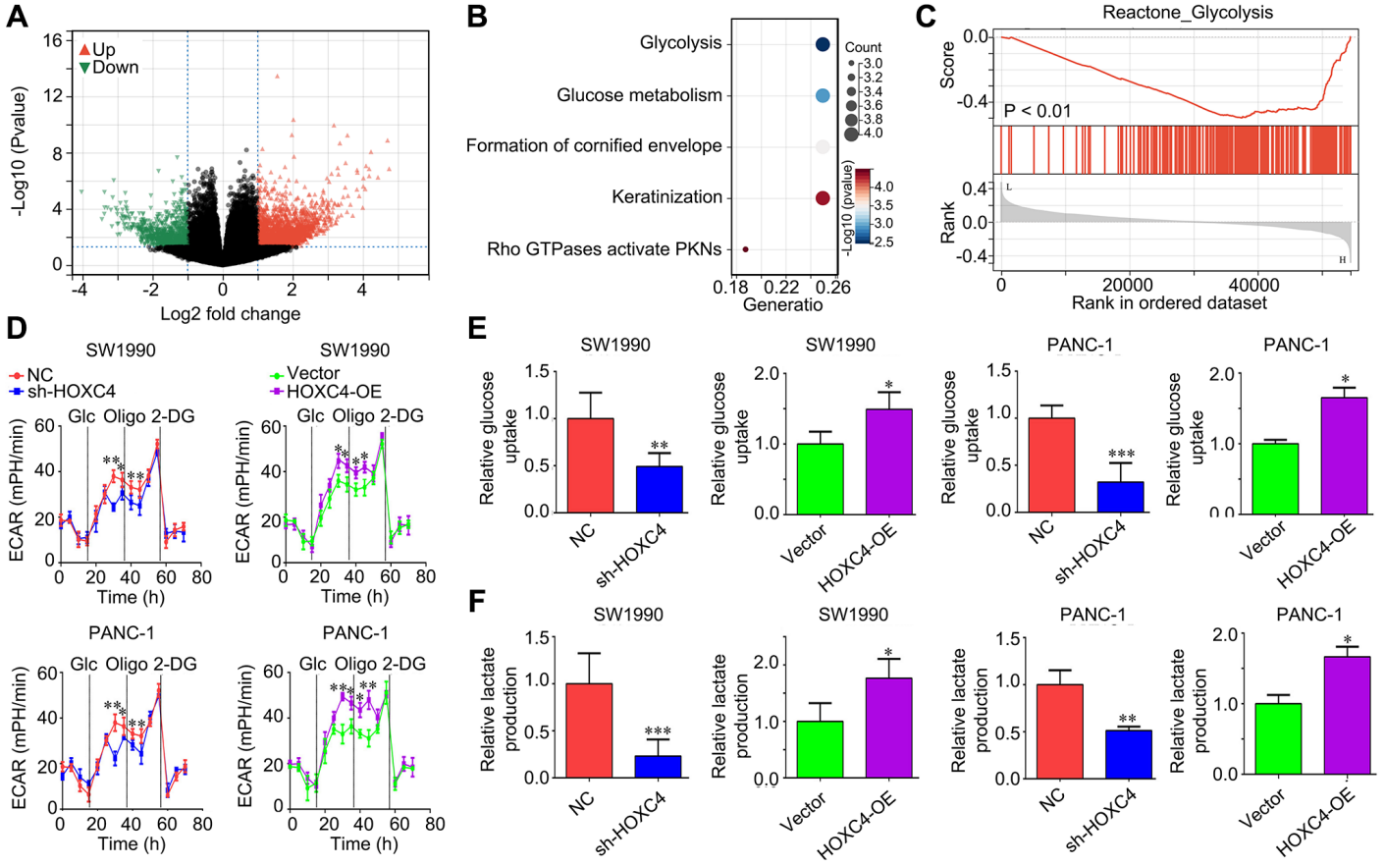
**HOXC4 regulates glycolysis in PC cells.** (**A**) DEGs between PC tissues with high and low HOXC4 expression. (**B**) Reactome analysis of the top200 up-regulated genes in PC with high HOXC4 expression. (**C**) GSEA for the expression of HOXC4. (**D**) Extracellular acidification rate in SW1990 and PANC-1 cells while HOXC4 was overexpressed or inhibited. (**E**, **F**) Glucose uptake and lactate production in SW1990 and PANC-1 cells while HOXC4 was overexpressed or inhibited. ^*^P < 0.05, ^**^P < 0.01.

In order to elucidate the molecular mechanisms of HOXC4 in PC, we subsequently stratified PC tissues from the TCGA cohort into two groups based on the median expression value of HOXC4: high expression and low expression. By conducting a differential gene expression analysis, we identified a total of 2488 up-regulated genes and 979 down-regulated genes in PC tissues with high HOXC4 expression compared to those with low HOXC4 expression (Figure 4A; Supplementary Table 2). After conducting Reactome analysis, it was observed that the top 200 up-regulated genes exhibited significant enrichment in various biological signaling, including “Glycolysis,” “Glucose metabolism,” “Formation of cornified envelope,” “Keratinization,” and “Rho GTPases activate PKNs” (Figure 4B; Supplementary Table 3). Furthermore, GSEA analysis revealed a correlation between HOXC4 expression and the activation of glycolysis (Figure 4C). Consequently, an investigation was conducted to determine the impact of HOXC4 on glycolysis in PC cells. The results of ECAR analysis demonstrated that HOXC4 knockdown led to a significant reduction in ECAR, whereas HOXC4 overexpression promoted it (Figure 4D). Furthermore, both glucose uptake and lactate production assays demonstrated that HOXC4 inhibition significantly suppressed glucose uptake and lactate production in PC cells, while overexpression of HOXC4 increased them (Figure 4E, 4F).

### HOXC4 directly promoted the transcription of LDHA

**Figure 5:**
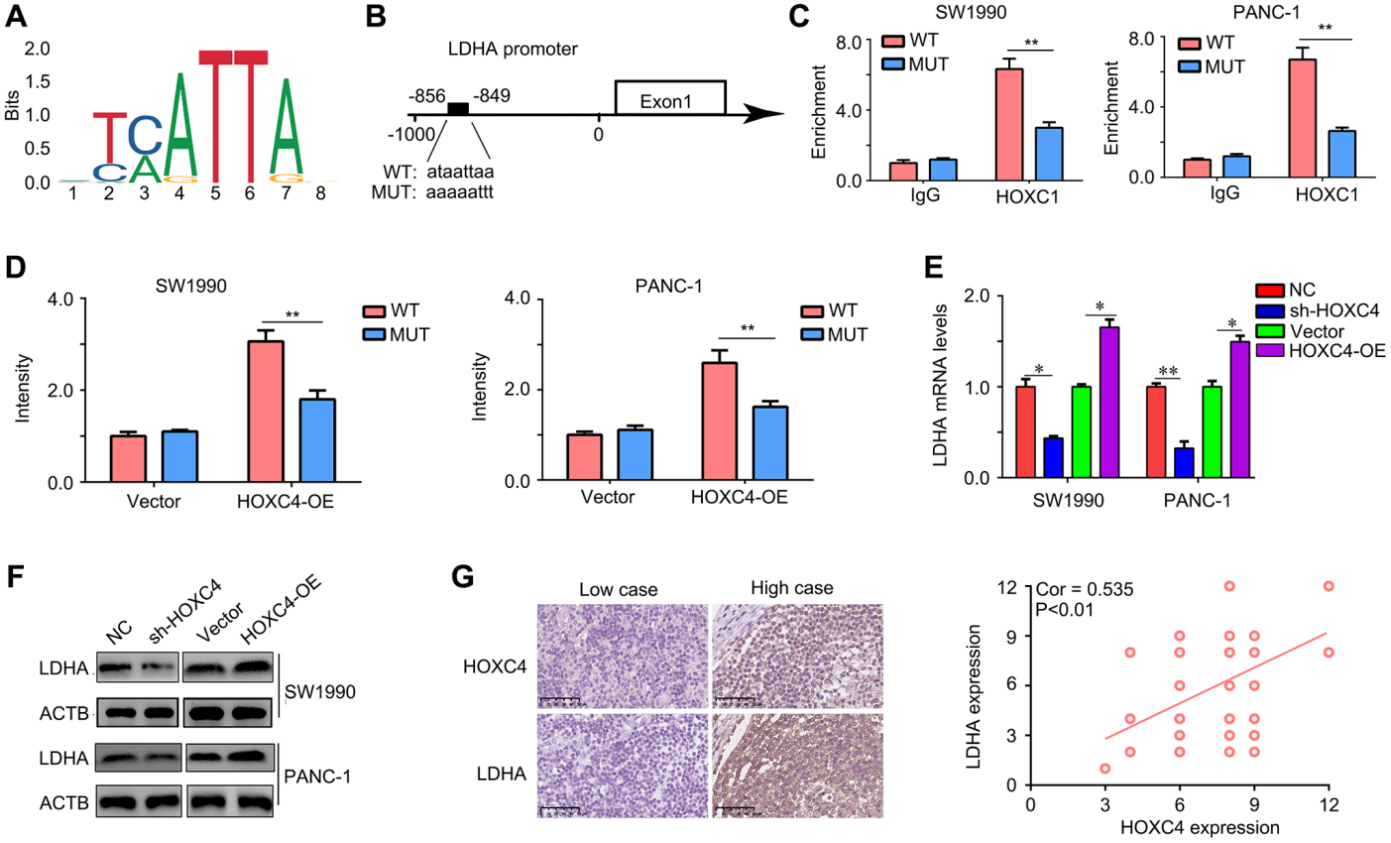
**LDHA is a target of HOXC4 in PC.** (**A**) Motif of HOXC4. (**B**) HOXC4 can bind to the promoter of LDHA. (**C**) Chip-qPCR was used to verify the binding. (**D**) HOXC4 increased the luciferase activity of LDHA in PC cells, while mutation of the associating element in the promoter of LDHA abolished the effect of the HOXC4 on luciferase activity. (**E**, **F**) The mRNA and protein expression of LDHA was regulated by HOXC4. (**G**) Co-expression between HOXC4 and LDHA in PC tissues. ^*^P < 0.05; ^**^P < 0.01.

In order to investigate the impact of HOXC4 on glycolysis, a bioinformatics analysis was conducted to examine the correlation between HOXC4 and crucial glycolytic enzymes. The binding motif of HOXC4 was initially obtained from the JASPAR database (https://jaspar.genereg.net/) (Figure 5A). Interestingly, it was discovered that HOXC4 has the ability to directly bind to the promoter region of a key glycolytic enzyme, namely LDHA (Figure 5B). The binding site was identified as −856 – −849 within the LDHA promoter (Figure 5B). To confirm the regulatory role of HOXC4 on LDHA, CHIP-qPCR was performed, which validated the binding interaction (Figure 5C). Luciferase report experiments indicated that HOXC4 overexpression can increased the fluorescence intensity in SW1990 and PANC-1 cells transfected with LDHA plasmids with wildtype promoter sequence, but no in those transfected with LDHA plasmids with mutation promoter sequence (Figure 5D). Moreover, we found that knockdown of HOXC4 significantly reduced the mRNA and protein levels of LDHA, while overexpression of HOXC4 increased their expression (Figure 5E, 5F). Furthermore, we found that LDHA expression was positively associated with HOXC4 expression in PC tissues (Figure 5G). Taken together, HOXC4 directly promoted the transcription of LDHA.

### Proliferation and glycolysis inhibiting effects of HOXC4 knockdown on PC cells were reversed by LDHA overexpression

**Figure 6:**
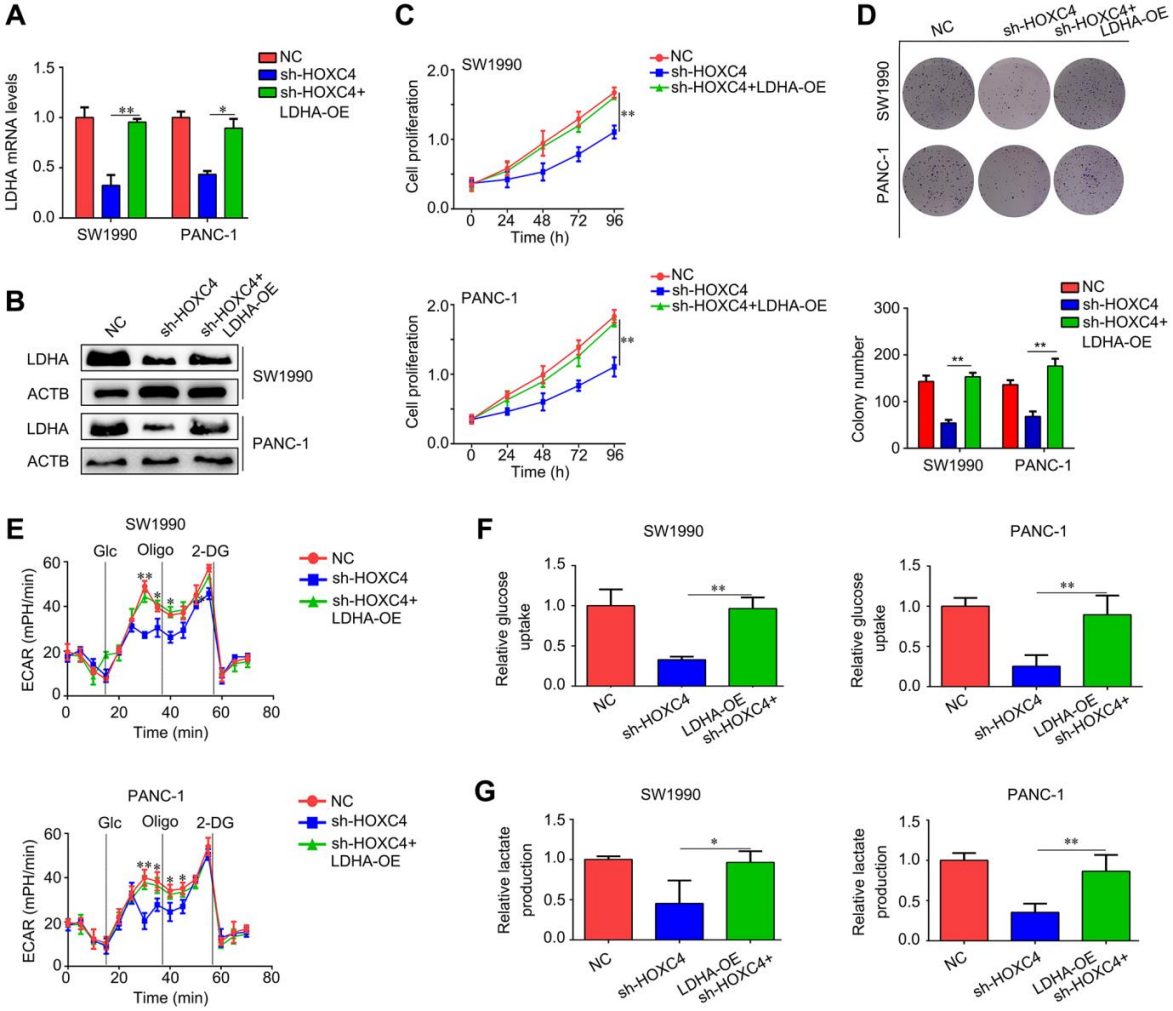
**LDHA overexpression decreases the inhibitory effect of HOXC4 knockdown on cell proliferation and glycolysis.** PC cells were divided into three groups, including normal control group (NC), HOXC4 knockdown group (sh-HOXC4) and cells with sh-HOXC4 and LDHA-overexpression. (**A**, **B**) Reverse transcription-quantitative PCR was used to detect the expression of LDHA in each group. (**C**) Cell count kit-8 assay was performed to detect the cell proliferation of each group. (**D**) Colony formation assay was performed to detect the colony formation of each group. (**E**) Analysis of the extracellular acidification rate for each group. (**F**, **G**) Glucose uptake and lactate production were analyzed in each group. ^*^P < 0.05, ^**^P < 0.01.

LDHA expression was restored in HOXC4-knockdown PC cells (Figure 6A, 6B). Based on the CCK-8 results, overexpression of LDHA diminished effects of HOXC4 knockdown on PC proliferation (Figure 6C). It was demonstrated by colony formation assays that LDHA overexpression abolished inhibitory effect of HOXC4 knockdown on PC cell colony formation (Figure 6D). Moreover, the results of ECAR analysis indicated that restoration of LDHA in HOXC4-knockdown PC cells significantly increased the ECAR (Figure 6E). Similarly, glucose uptake and lactate production analyses indicated that increasing the expression of LDHA in HOXC4-knockdown PC cells significantly increased their ability of glucose uptake and lactate production (Figure 6F, 6G). In conclusion, overexpression of LDHA reduced the inhibitory effects of HOXC4 knockdown on PC cell proliferation and glycolysis.

## DISCUSSION

In the current study, we demonstrated that HOXC4 was elevated in PC tissues, and positively associated with poor prognosis. Knockdown of HOXC4 significantly reduced proliferation and glycolysis in PC cells, as well as inducing cell apoptosis and G1 phase arrest. Mechanically, we demonstrated that HOXC4 can directly bind to the promoter of LDHA and increase its expression, thus inducing the enhancement of proliferation and glycolysis of PC cells. Our study emphasized that HOXC4 was an oncogene in PC, which linked PC progression and glycolysis.

The HOX family is a class of transcription factors widely distributed in cells. Dysregulation of HOXs in PC cell had been widely studied. Fox example, HOXA10 was found to elevate in PC tissues, and associated with poor prognosis [19]. Elevated HOXB13 in PC tissues predicted more metastasis cases [20]. HOXA1 was regulated by miR-10a in PC tissues and had potential to promote PC cell invasiveness [21]. However, the role of HOXC4 in PC was still known limitedly. Our study provided the first evidence that HOXC4 was an oncogene in PC, and it links to PC cell proliferation and glycolysis, indicating that HOXC4 may be a potential target for PC therapy.

Various studies have demonstrated that cancer cells tend to metabolize energy using glycolysis even in oxygen-rich environments, which can produce more ATP in a short time [22]. LDHA is a key gene regulating the process of glycolysis, which is commonly upregulated in cancer tissues [23, 24]. LDHA was upregulated in oral squamous cell carcinoma and promoted cancer progression by enhancing glycolysis [25]. LDHA was increased under hypoxia and promoted the development of bladder cancer [26]. LDHA was overexpressed in PC tissues and predicted shorter survival days [27]. Inhibition of LDHA decreased cell proliferation and migration by suppressing glycolysis [28]. In the current study, we provided the first evidencee that HOXC4 was linked to glycolysis while it can bind to increase LDHA transcription. Therefore, HOXC4 may be a key target to inhibit glycolysis of PC cells.

As a result of the present study, HOXC4, which is increased in PC, may be a tumor promoter for the progression of this disease. HOXC4 increased the proliferation of PC cells via promoting LDHA-mediated glycolysis. HOXC4 may contribute to the diagnosis and therapy of PC as a novel biomarker and target. Actually, the clinical value of HOXC4 still needed to be validated in larger samples.

## MATERIALS AND METHODS

### Clinical samples

PC and adjacent tissues were gained from 60 patients (male/female = 41/43; age, 43–76 years) undergoing surgery for PC at The Affiliated Hospital of Guizhou Medical University. Radiation therapy or chemotherapy were not administered to any patients before surgery. In accordance with the Declaration of Helsinki, tissues collection was approved by the Ethics Committee of Guizhou Medical University (Approved Number: 2019LS78). Written informed consent was provided by all participants.

### Cell culture and transfection

The source of PC cell lines (SW1990 and PANC-1) was American Type Culture Collection (USA). Cell culture was performed at 37°C in DMEM (HyClone, USA; Cytiva) containing 10% FBS (HyClone; Cytiva) and 5% CO_2_. HOXC4 overexpression lentiviruses, negative control (NC) lentiviruses of HOXC4, HOXC4 shRNAs and NC shRNAs were gained from Sangon Biotech Co., Ltd. (China). GeneCopoeia, Inc. (USA) provided the overexpression plasmid of LDHA and the empty plasmid. The transfection process was performed using Lipofectamine 2000 (Invitrogen, USA) as directed by the manufacturer. Targeting HOXC4 shRNAs was shown as follows: sense sequence, 5′-GGUUCAAAUAUGUAACUAA-3′, and antisense sequence, 5′-UUAGUUACAUAUUUGA ACC-3′.

### RT-qPCR

We extracted total RNA from PC cells and tissues using TRIzol^®^ reagent (Takara Bio, Inc., Japan). Using PrimeScript^™^ RT Reagent kit (Takara Bio, Inc.) for mRNA, cDNA was synthesized. By using SYBR Green reagent (Sangon Biotech Co., Ltd.), HOXC4 and LDHA expression levels were measured. ACTB was set as loading control. Primer sequences are exhibited as follows: HOXC4 forward sequence, 5′-GAGCGCC AGTATAGCTGCAC-3′; HOXC4 reverse sequence, 5′-GAGCGCCAGTATAGCTGCAC-3′; LDHA forward sequence, 5′-ATGGCAACTCTAAAGGATCAGC-3′; LDHA reverse sequence, 5′-CCAACCCCAACAACT GTAATCT-3′; β-actin forward sequence, 5′-CAT GTACGTTGCTATCCAGGC-3′; and β-actin reverse sequence, 5′-CTCCTTAATGTCACGCACGAT-3′.

### Immunohistochemistry (IHC)

A graded xylene and ethanol solution was used to dewax and rehydrate the paraffin-embedded PC tumor tissues. Followed by utilizing sodium citrate for antigen retrieval and H_2_O_2_ for blocking endogenous peroxidase activity, the tissue samples were incubated with antibodies against HOXC4 (1:50; Cat No. PA5-67617; Thermo Fisher Scientific, USA), Ki67 (1:100; Cat No. A20018; Abclonal, Inc., USA) and PCNA (1:100; Cat No. A13336; Abclonal, Inc.) overnight at 4°C. The PC tissues were washed with PBS before being incubated with secondary antibodies (1:200; Shanghai Univ Biological Technology, Ltd.) and stained with 3,3′-diaminobenzidine and hematoxylin. The images were captured with a light microscope imaging system (Olympus Corporation, Japan, 400X). For gene expression of HOXC4, HOXC4 expression in PC tissues/normal tissues ≥1.5, 1.5–1.0 and 1.0–0.667 was set as significantly increasing, non-significantly increasing and non-significantly reducing, especially.

### Cell counting kit-8 (CCK-8) assay

In 96-well plates, SW1990 and PANC-1 cells were seeded at a density of 5 × 10^3^ cells per well, and each group consisted of six replicates. Briefly, each well was injected with 10 μl CCK-8 reagent (Shanghai Univ Biological Technology, Ltd.) at 24, 48, 72 and 96 h. By using an automatic enzyme label detector (BioTek, USA), the absorbency of PC cells was determined at 450 nm for each well.

### Colony formation assay

In total, six-well plates containing 500 SW1990 or PANC-1 cells were cultured at 37°C for 14 days. Subsequently, we discarded the medium, fixed the colonies with 4% paraformaldehyde (Beyotime Institute of Biotechnology, China) for 15 minutes, stained them with crystal violet for 20 minutes, and numbered them.

### Flow cytometry analysis

For apoptosis detection, it was conducted with the Annexin V/PI Apoptosis Detection kit (Thermo Fisher Scientific, Inc.) in SW1990 and PANC-1. For cell cycle distribution analysis, a total of two washes with PBS were conducted on SW1990 and PANC-1 cells followed by a 24-hour fixation with 75% ethanol in 4°C. Afterwards, the cells were treated with a PI solution at 37°C for 20 minutes after ethanol removal. The percentage of apoptotic cells and cell cycle distribution were detected by Attune NxT flow cytometry (Thermo Fisher Scientific, Inc.), and Flow Jo software (version: 7.4; FlowJo LLC, USA) was used to analyze the results.

### Western blotting

Using RIPA lysis buffer (Shanghai Univ Biological Technology, Ltd., China) having 5% PMSF protease inhibitor (Shanghai Univ Biological Technology, Ltd.), we isolated total protein from PC cells and tissues. Protein concentrations were measured by a BCA method. Proteins (30 μg per lane) were separated by 10% SDS-PAGE for 120 min. A 0.22-μm diameter polyvinylidene fluoride membrane (EMD Millipore, USA) was then used to transfer the proteins. A 5% non-fat milk was used for blocking the membranes, followed by incubation for 12 hours at 4°C with primary antibodies against HOXC4 (1:500; Cat No. PA5-67617; Thermo Fisher Scientific), Bcl-2 (1:1000; Cat No. 12789-1-AP; ProteinTech Group, Inc., China), Bax (1:1000; Cat No. 50599-2-Ig; ProteinTech Group, Inc.), caspase 3 (1:1000; Cat No. 19677-1-AP; ProteinTech Group, Inc.), cyclin-dependent kinase 1 (CDK1; 1:1000; Cat No. 19532-1-AP; ProteinTech Group, Inc.), cyclin D1 (1:1000; Cat No. 26939-1-AP; ProteinTech Group, Inc.), cleaved caspase 3 (1:2000; Cat no.9664; Cell Signaling Technology, Inc., USA), MMP-2 (1:1000; Cat No. 10373-2-AP; ProteinTech Group, Inc.), MMP-9 (1:1000; Cat No. 10375-2-AP; ProteinTech Group, Inc.), LDHA (1:1000; Cat No. 19987-1-AP; ProteinTech Group, Inc.) and β-actin (1:1000; Cat No. 20536-1-AP; ProteinTech Group, Inc.). After culturing the secondary antibodies (1:5,000) for 2 h, the blots were visualized in a MultiImager system (Bio-Rad Laboratories, Inc., USA) using a high-sensitivity ECL reagent (Boster Biological Technology, China) and ImageJ (version: 1.51) was taken for calculating protein relative expression. β-actin was a reference for normalization of HOXC4, Bcl-2, Bax, CDK1, cyclin D1, MMP-2, MMP-9 and LDHA, while caspase 3 was used for the normalization of cleaved caspase 3.

### Subcutaneous tumorigenesis model

In accordance with the Chinese Guidelines for Ethical Review, the Guizhou Medical University Ethics Committee approved the animal experiment (approved number: 2100616). In total, female BALB/c nude mice (*N* = 10) were gained from the Animal Center of Guizhou Medical University. Following by adaptive feeding in specified pathogen free environment, 200 μl PBS with a density of 5 × 10^6^/ml PANC-1 cells which transfected with NC or sh-HOXC4 were subcutaneously injected into the right upper flank of mice. Health statuses were monitored on a daily basis, while tumor volume was measured on a weekly basis, and calculated with the following formula: Tumor volume (mm^3^) = (long diameter × width^2^)/2. The mice were anesthetized with pentobarbital at a dose of 60 mg/kg injected intravenously while the volume of tumor tissues reached near 600 mm^3^, and euthanized using a cervical dislocation method. As a final step, the tumor tissues were extracted and tested for Ki67 and proliferating cell nuclear antigen (PCNA) expression.

### Detection of extracellular acidification rate (ECAR), glucose uptake and lactate production

ECAR detection of SW1990 and PANC-1 cells was performed with XF24 extracellular analyzer (Seahorse Bioscience, USA). The 24-well cell culture plates were seeded with SW1990 and PANC-1 cells at a density of 2 × 10^4^ cells per well. For ECAR detection, glucose, oligomycin A (an inhibitor of oxidative phosphorylation; MCE, Wuhan, China) and 2-deoxy-d-glucose (an inhibitor of glycolysis; 2-DG; MCE, Wuhan) were added after analyzer calibration. Normalization of results was performed based on protein concentration. The 96-well plates were seeded with 3 × 10^3^ SW1990 and PANC-1 cells for detection of glucose uptake. After removing the medium, Krebs-Ringer phosphate HEPES buffer containing 5% BSA (Shanghai Univ Biological Technology, Ltd.) was added. Subsequently, cells in each well were treated with 2-DG (10 mM). Glucose uptake was measured with a Glucose Uptake Colorimetric Assay kit (Sigma-Aldrich, USA) following the manufacturer’s instructions. For lactate production detection, SW1990 and PANC-1 were lysed and homogenized using RIPA, and the Lactate Assay Kit II (Shanghai Univ Biological Technology, Ltd.) was conducted in accordance with the manufacturer’s instructions.

### Dual luciferase reporter assay

Following analysis of LDHA as a target gene of HOXC4 using the JASPAR, the binding effects was verified using a dual luciferase reporter assay. LDHA promoter wild-type (Wt) or mutant (Mut) sequences were ligated into psiCHECK-2 reporter vectors (Promega Corporation, USA). Subsequently, Lipofectamine^®^ 2000 (Beijing Solarbio Science and Technology Co., Ltd., China) was used to transfect the Wt/Mut LDHA promoter luciferase reporter vector into PC cells with HOXC4 overexpression and NC cells. Finally, the luciferase activity of the cells was determined following transfection at 24 h.

### Statistical analysis

SPSS 20.0 (IBM Corporation) was conducted to analyze all results (*n* = 3; mean ± SD) in the current study. Two groups were compared using a twin-tail Student’s *t*-test. Multiple groups were compared using ANOVA followed by Bonferroni’s post hoc test. *P* < 0.05 was significant.

### Availability of data and materials

The results used and/or analyzed in the current study are available from the corresponding author upon reasonable request.

## Supplementary Materials

Supplementary Tables 1 and 3

Supplementary Table 2
