# Supplementary material for: HOXC4 promotes proliferation of pancreatic cancer cells by increasing LDHA-mediated glycolysis
Source: Aging (Albany NY). 2024 Jul 9;16(13):11103–16. doi: 10.18632/aging.206008 (PMC11272123; doi:10.18632/aging.206008)
Supplement: Supplementary Tables 1 and 3 [file aging-16-206008-s001.pdf]

SUPPLEMENTARY TABLES

Supplementary Table 1. Member of HOX family was used for analysis.

HOX family number
HOXA1
HOXA10
HOXA11
HOXA13
HOXA2
HOXA3
HOXA4
HOXA5
HOXA6
HOXA7
HOXA9
HOXB1
HOXB13
HOXB2
HOXB3
HOXB4
HOXB5
HOXB6
HOXB7
HOXB8
HOXB9
HOXC10
HOXC11
HOXC12
HOXC13
HOXC4
HOXC5
HOXC6
HOXC8
HOXC9
HOXD1
HOXD10
HOXD11
HOXD12
HOXD13
HOXD3
HOXD4
HOXD8
HOXD9

Supplementary Table 3. Terms of top 200 up-regulated genes in HOXC4 high PC tissues enriched in various biological signaling.

Terms	Gene ratio	*P*-value	Gene
GLYCOLYSIS	4/16	2.92707E-06	PKM/HK2/PFKP/LDHA
GLUCOSE_METABOLISM	4/16	7.81135E-06	PKM/HK2/PFKP/LDHA
FORMATION_OF_THE_CORNIFIED_ENVELOPE	4/16	2.97923E-05	SPRR1A/KRT3/KLK8/SPRR3
KERATINIZATION	4/16	0.000225009	SPRR1A/KRT3/KLK8/SPRR3
RHO_GTPASES_ACTIVATE_PKNS	3/16	0.00031488	KLK2/H2AC14/KLK3
